# Masquerade syndrome in ocular surface squamous neoplasia

**DOI:** 10.5935/0004-2749.20230027

**Published:** 2023

**Authors:** Raysa Victoria de Oliveira Cechim, Laura Caldas dos Santos, Dalton de Freitas Santoro, Luiz Antônio Vieira, Luciene Barbosa de Sousa, Denise de Freitas

**Affiliations:** 1 Cornea and External Eye Disease Program, Department of Ophthalmology and Visual Sciences, Escola Paulista de Medicina, Universidade Federal de São Paulo, São Paulo, SP, Brazil; 2 Department of Ophthalmology and Visual Sciences, Escola Paulista de Medicina, Universidade Federal de São Paulo, São Paulo, SP, Brazil; 3 Escola Paulista de Medicina, Universidade Federal de São Paulo, São Paulo, SP, Brazil

**Keywords:** Neoplasms, squamous cell, Conjunctival neoplasms, Corneal diseases, Eye neoplasms, Scleritis, Interferon alpha-2, Neoplasias de células escamosas, Neoplasias da túnica conjuntiva, Doenças da córnea, Neoplasias oculares Esclerite, Interferon alfa-2

## Abstract

The aim of this study was to alert the ophthalmic community to an atypical manifestation of ocular surface squamous neoplasia, which may delay diagnosis and treatment and result in a guarded visual prognosis and significant sequelae. A 61-year-old immunocompetent man presented with an initial diagnosis of necrotizing scleritis in the right eye for 3 months. He was treated with systemic prednisone but experienced persistent pain and low visual acuity. Conjunctival biopsy of the affected region confirmed the diagnosis of invasive ocular surface squamous neoplasia, which progressed with intraocular and orbital invasion; thus, exenteration was performed. Masquerade syndrome should be suspected in patients with nodulo-ulcerative lesions of the conjunctiva and sclera. This clinical can be more aggressive, with a greater likelihood of intraocular and orbital involvement. The earlier the diagnosis and treatment, the better the patient prognosis.

## Abstract (pt)

O objetivo é alertar a comunidade oftalmológica sobre uma manifestação atípica de neoplasia escamosa da superfície ocular (*OSSN*) que pode levar a um atraso no diagnóstico e tratamento, evoluindo com prognóstico reservado e significativas sequelas. Homem, imunocompetente, 61 anos com diagnóstico inicial de esclerite necrosante em olho direito há 3 meses, em tratamento com prednisona sistêmica porém com persistência da dor e baixa acuidade visual. Realizado biópsia conjuntival em região acometida e diagnosticado como neoplasia escamosa da superfície ocular invasiva. Evolui com invasão intraocular e orbital sendo submetido a exenteração. Assim sendo, deve-se suspeitar de síndrome mascarada frente a um paciente com lesões nódulo-ulcerativas da conjuntiva e esclera. Essa forma clínica pode ser mais agressiva, com maior chance de comprometimento intraocular e orbital. Quanto mais precoces o diagnóstico e o tratamento, melhor o prognóstico para o paciente.

## INTRODUCTION

Ocular surface squamous neoplasia (OSSN) is the most frequent non-pigmented tumor of the ocular surface. It encompasses a spectrum of neoplastic changes in the squamous epithelium of the cornea and conjunctiva that range in severity from epithelial dysplasia to intraepithelial neoplasia *in situ* to invasive squamous cell carcinoma^(1)^. It affects mainly men in the sixth decade of life who live in areas of greater sun exposure^(2)^. The lesions typically present unilaterally as a gelatinous mass in the region of the limbus and in the sun-exposed interpalpebral fissure (medially or laterally). Other common forms include leukoplakic, opalescent, papillomatous lesions, with irregular borders and/or dilated and tortuous vessels, which may invade the adjacent corneal epithelium^(1)^. Cases of masquerade syndrome can occur in OSSN, which leads to delayed diagnosis and possible worsening of patient prognosis. Misdiagnosis has been reported in the literature, including upper limbic conjunctivitis, blepharitis, peripheral ulcerative keratitis, and pterygium^(3)^. The aim of this case report was to alert the ophthalmic community to another atypical manifestation of ocular surface squamous neoplasia (OSSN), which may lead to delayed diagnosis and treatment and result in a guarded visual prognosis and significant sequelae.

## CASE REPORT

**Figure 1:**
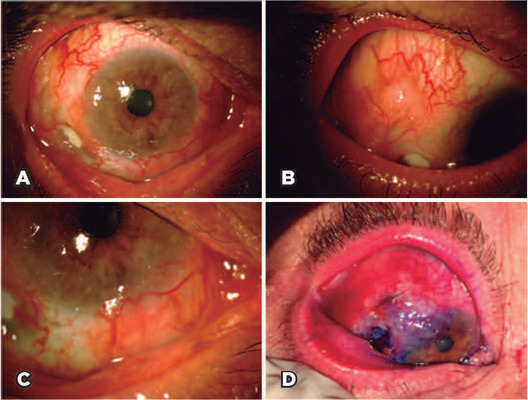
Intense difuse conjunctival hyperemia, engorgement of the conjunctival and deeper vessels with loss of normal anatomy (A); conjunctival nodule (B); inferior temporal scleral thinning and melting (C); and ocular surface stained with 1% toluidine blue, highlighting the afected areas in the sclera and conjunctiva (D).

A 61-year-old immunocompetent man with a well-controlled diabetes presented with a complaint of pain in the right eye for 6 months, which progressed to low visual acuity for 2 months. He reported treatment with oral prednisone for 3 months for necrotizing scleritis, without improvement and negative serological and laboratory test results for systemic diseases. His best-corrected visual acuity was 20/50 in the right eye and 20/20 in the left eye. Previous biomicroscopy revealed intense diffuse conjunctival hyperemia, engorgement of the conjunctival and deeper vessels with loss of normal anatomy, inferior temporal scleral thinning and melting, conjunctival nodule, and transparent cornea with a discrete inferior nasal perilimbal gelatinous lesion. Ocular surface staining with 1% toluidine blue highlighted the affected areas in the sclera and conjunctiva (Figure 1).

**Figure 2:**
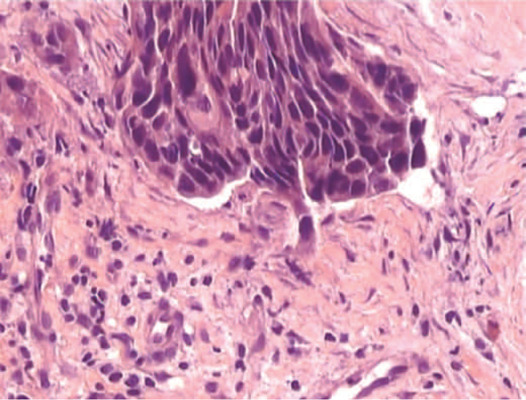
Histopathologic fndings compatible with OSSN.

The presumptive diagnosis was masquerade syndrome secondary to neoplasia, confirmed by an incisional biopsy of the bulbar conjunctiva that revealed moderately differentiated invasive OSSN (Figure 2). Orbital magnetic resonance imaging (MRI) and ultrasonographic biomicroscopy ruled out intraocular involvement and/or orbital invasion. The patient started receiving subconjunctival interferon alpha-2b at 3 million IU/mL weekly combined with topical interferon 1 million IU/mL 4 times a day for the extensive conjunctival tumor, although it did not affect the eyelid or orbit.

One month after starting the treatment, the patient showed improvements of the lesions and scleral thinning but worsening of the pain. A second MRI revealed thickening and infiltration of the inferior-posterior sclera and inferior orbital structures. Ocular ultrasonography confirmed the MRI findings and additionally revealed a T-sign suggestive of posterior scleritis secondary to the presence of a tumor mass. Given the evidence of tumor invasion, exenteration was performed. Histopathological examination confirmed the tumor extension affecting the periorbital adipose and muscle tissue, optic nerve, inferior bulbar and tarsal conjunctiva, sclera, and cornea, and infiltrating the intraocular structures of the anterior and posterior chambers. No metastases have been detected to date.

## DISCUSSION

Six articles in the literature had a total of 10 case reports on scleritis such as masquerade syndrome in OSSN (Table 1). In all the case reports, the patients were male, with a mean age of 53 years (range, 31-76 years)^(3,4,5,6,7,8)^. This is similar to our case of a 61-year-old man and consistent with the general data on OSSN, which prevails among elderly men^(1)^.

**Table 1:** Characteristics of cases of ocular surface squamous neoplasia (OSSN) misdiagnosed as necrotizing scleritis

Reference	Age (years)	Sex	General health	∆t Dx (months)	Treatment	OSSN differentiation
Lindenmuth et al.^4^	64	M	Good	12	Exenteration	NR
Kim et al.^5^	61	M	HIV +	0.5	Cryotherapy	NR
Younan and McClellan^6^	31	M	Good	4	Surgical excision	Well differentiated
Mahmood et al.^7^	76	M	Good	24	Enucleation	Well differentiated
	61	M	NR	9	Enucleation	NR
Sharma et al.^8^	35	M	Good	6	Enucleation	Well differentiated
Kaliki et al.^3^	56	M	Good	3	Exenteration	Well differentiated
	42	M	Good	3	Exenteration	Well differentiated
	52	M	HIV +	5	Exenteration	Moderately differentiated
	56	M	Good	12	Exenteration	Mucoepidermoid
Present report	61	M	Good	6	Exenteration	Moderately differentiated

M= male; HIV= human immunodeficiency virus; NR= not reported; ∆*t* Dx= delay in diagnosis; OSSN= ocular surface squamous neoplasia.

Immunosuppression, especially in human immunodeficiency virus (HIV)-positive patients; organ transplant; and autoimmune diseases are risk factors of OSSN, which may result in more aggressive and atypical presentations, with worse outcomes^(1,9)^. Of the cases reported in the literature comparable with ours, only two patients had HIV infection^(6,7)^; all the others, including the present patient, were immunocompetent and had no significant comorbidities. The literature also describes the occurrence of OSSN in an immunosuppressed patient taking systemic corticosteroids and in patients treated with topical corticosteroids after corneal transplant^(10)^. Although our patient did not use corticosteroids for a prolonged period, his intake of the drugs might have contributed to the more aggressive course of neoplasia.

The initial presentation of the cases in the literature and our case can be highlighted, as it is not usually related to OSSN gelatinous mass in the limb region^(1)^. The manifestations in almost all reported cases were similar to those in our case^(3,4,5,6,7,8)^, drawing attention to conjunctival nodules that can be mistaken for nodular scleritis, ulcers, and conjunctival-scleral necrosis, which is often initially misdiagnosed as necrotizing scleritis^(3)^. Patients with scleritis, except for scleromalacia perforans, have significant pain, but those with masquerade syndrome do not have pain. When pain is present, mistakenly interpreted as scleritis, it occurs only in the stages of scleral invasion in patients with OSSN, which should prompt further investigation^(3)^.

In most case reports, including ours, the patients underwent screening for systemic infectious and rheumatic diseases, without identifying pathologies; some patients underwent treatment with topical and/or systemic corticosteroids^(5,6,8)^, which delayed accurate diagnosis and specific treatment for neoplasia. Owing to the progression and/or non-response of the disease, the patients underwent biopsy to confirm the diagnosis of neoplasia. The time between disease onset and OSSN diagnosis ranged from 0.5 to 24 months. As a consequence, this delay can be related to worse prognosis.

The treatment of invasive squamous cell carcinoma usually consists of total excision and cryotherapy of the tumor bed^(5)^. However, the nodulo-ulcerative variant shows an aggressive clinical and morphological pattern with a high incidence of scleral/tarsal (100%) and intraocular (67%) or orbital (33%) tumor extension^(3)^. In cases of conjunctival and scleral ulceration, a high index of suspicion and aggressive treatment with extended enucleation or subtotal orbital exenteration can be indicated. In cases of conjunctivo-tarsal ulceration, wide excision and extended eyelid reconstruction are recommended^(3)^. In our case, to maintain the eyeball, primary chemotherapy was indicated because of the large extent of the lesion. At first, our patient showed clinical improvement with the treatment, but after he developed severe pain, further investigation revealed progression of the neoplasm with involvement of the intraocular and orbital structures, thus requiring exenteration. Previous reports also described intraocular and/ or orbital involvement, which likewise required enucleation or exenteration, demonstrating the aggressiveness of this type of tumor presentation. Similar to our case, the previous cases showed no metastasis.

Invasive squamous cell carcinoma of the conjunctiva and intraocular invasion are rare. They occur in approximately 13% of cases and are closely related to aggressive histological variants (mucoepidermoid, spindle-cell, and adenoid) and/or to previous surgical intervention^(3,5,9)^. Although most reported cases showed well-differentiated neoplasms, theoretically with more indolent behavior^(6)^, the guarded prognosis was probably due to a significant delay in diagnosis (from 2 weeks to 12 months)(^9)^. This allowed the disease to progress and a possible tendency of endophytic tumor growth in cases of scleral melting and surgical manipulation^(8)^. According to Mahmood et al., intraocular invasion is believed to occur owing to a combination of factors such as tumor growth associated with intense inflammation with consequent destruction of ocular tissues and vascular invasion^(5)^. Diffuse growth with inflammation and vascular invasion seems to contribute to thinning, necrosis, and perforation of the ocular tissues, resulting in intraocular spread even without a tumor mass^(5)^.

The first case in the literature was reported in 1988, recent cases were reported in 2017, and our case was reported in 2020, proving that the ophthalmic community is not fully aware of this issue. In conclusion, ocular inflammation in the presence of conjunctival ulceration, nodule formation, scleral thinning, and/or necrosis without identification of a systemic cause and that does not respond to conventional treatment should promptly raise the hypothesis of masquerade syndrome. As noted in the present case report, this nodulo-ulcerative form of OSSN is more aggressive, with greater intraocular and orbital involvements. Therefore, the earlier the diagnosis and treatment, the better the patient prognosis.
